# Microstructure Dependence of Effective Thermal Conductivity of EB-PVD TBCs

**DOI:** 10.3390/ma14081838

**Published:** 2021-04-07

**Authors:** Shi-Yi Qiu, Chen-Wu Wu, Chen-Guang Huang, Yue Ma, Hong-Bo Guo

**Affiliations:** 1 Beijing Key Laboratory for Advanced Functional Material and Thin Film Technology, School of Materials Science and Engineering, Beihang University (BUAA), Beijing 100191, China; qiusy@buaa.edu.cn (S.-Y.Q.); guo.hongbo@buaa.edu.cn (H.-B.G.); 2 Institute of Mechanics, Chinese Academy of Sciences, Beijing 100190, China; huangcg@imech.ac.cn

**Keywords:** thermal barrier coatings, electron beam physical vapor deposition, thermal conductivity, finite element simulation

## Abstract

Microstructure dependence of effective thermal conductivity of the coating was investigated to optimize the thermal insulation of columnar structure electron beam physical vapor deposition (EB-PVD coating), considering constraints by mechanical stress. First, a three-dimensional finite element model of multiple columnar structure was established to involve thermal contact resistance across the interfaces between the adjacent columnar structures. Then, the mathematical formula of each structural parameter was derived to demonstrate the numerical outcome and predict the effective thermal conductivity. After that, the heat conduction characteristics of the columnar structured coating was analyzed to reveal the dependence of the effective thermal conductivity of the thermal barrier coatings (TBCs) on its microstructure characteristics, including the column diameter, the thickness of coating, the ratio of the height of fine column to coarse column and the inclination angle of columns. Finally, the influence of each microstructural parameter on the mechanical stress of the TBCs was studied by a mathematic model, and the optimization of the inclination angle was proposed, considering the thermal insulation and mechanical stress of the coating.

## 1. Introduction

Thermal barrier coatings (TBCs) are applied on the surface of superalloy substrates to reduce the working temperature of the superalloy substrate and avoid high temperature oxidation wear and corrosion [1,2,3]. A classical thermal barrier coating structure is composed of a ceramic coat and bonding coat. The former is mainly for thermal insulation, while the latter is to relieve the stress caused by thermal expansion mismatch between the superalloy and ceramic coat [4,5,6,7]. In order to optimize the performance of the ceramic coating, coatings with different microstructures can be obtained by utilizing different processes. Electron beam physical vapor deposition (EB-PVD), an advanced thermal barrier coating preparative technique, works by electron beam bombardment making the target material melt quickly, evaporate and deposit on the surface of the substrate to form the coating. [8,9,10] During the formation of the coating, gas molecules are continuously deposited on the substrate, and the coating has a unique columnar microstructure, which has a high strain tolerance and long thermal cycle life. However, due to the lack of transverse discontinuous interface impeded heat flow in the column microstructure, the thermal insulation performance of the EB-PVD coating is not good enough [11,12,13,14].

For the sake of enhancing the thermal insulation performance of a columnar structure EBPVD coating, many scholars have carried out experimental methods to investigate the effect of microstructure on the thermal conductivity of EB-PVD coatings. Singh [15] utilized the method of interrupting the deposition process to introduce transverse interfaces into multiple layers of the EB-PVD coating, which enhanced phonons scattering as well as photons reflection, thus greatly reduced the heat transfer rate, and lowering the thermal conductivity of coatings. Nicholls [16] claimed the addition of dopant atoms in a ceramic material played a role in reducing ‘phonon’ transport [17,18,19]; the layered microstructures of EB-PVD coating were effective in reducing ‘photon’ transport [20,21,22], and the nanograined structures could diminish both phonons and photons transport [17,23], which could lower the thermal conductivity of coatings. H.-J. Rätzer-Scheibe et al. [24] studied the dependence of thermal conductivity on the thickness of EB-PVD thermal barrier coatings, and the results indicated that thermal conductivity of the thermal barrier coating would increase with thickness of the coating increasing. Renteria et al. [25] varied the EB-PVD process parameters to gain different morphology coatings to investigate the effect of morphology on the thermal conductivity of EB-PVD coatings. The results showed that the thermal conductivity of these coatings was affected by the inner-column pores as well as intercolumn open pores, and that the surface-area for open pores decreased as the diameter of the column increased. Gu et al. [26] used EB-PVD to synthesize a kind of highly porous thermal barrier coating with zig-zag morphology pores which could resist longitudinal heat flow and claimed this kind of zig-zag intercolumn gap could diminish the thermal conductivity by more than half. Singh [27] and Josell [28] prepared nanoscale multilayered EB-PVD thermal barrier coatings with component of Al_2_O_3_ and YSZ, and Jang [29,30] prepared nanoporous multilayer EB-PVD coatings and studied the heat conduction behavior of the nanomicrostructure of columnar structure coatings.

Meanwhile, theoretical and numerical methods have been widely used in the study of thermophysical properties of TBCs. Lu and Gu [26] conducted a combined analytical/numerical research of thermal conduction of the EB-PVD coating with zig-zag morphology pores, and investigated the dependence of thermal conductivity on morphologic parameters of zig-zag pores of the coating. Wu and Qiu [31] proposed a finite element coating model with discontinuous interfaces to reveal the dependence of the thermal insulation property on interlayer discontinuity of thermal barrier coatings. H.-J. Ra¨tzer-Scheibe et al. [24] presented an analytical model of the columnar structure coating to calculate the thermal conductivity of the coating, in which thermal conductivity was equal to the thermal conductivity of the inner zone with fine grained columns plus the thermal conductivity of the outer zone with coarse grained columns. Ganvir [32] set up an object-oriented finite element model of a columnar structure coating to analyze its thermal conductivity and claimed that microscale pores and coarse vertical cracks both played critical role in diminishing the thermal conductivity of the coating. Wang [33] used the Lattice Bolzmann method (LBM) to develop a model to study heat conduction behaviors in thermal barrier coatings, and the effect of porosity on thermal conductivity of TBCs. In this model, the four parameters stochastic growth method was adopted to rebuild the microstructure of the coating. However, there was no description of the detailed columnar structure to study the heat conduction characteristics of multiple adjacent columns. This is a significant factor to optimize columnar microstructure of EB-PVD TBCs with low thermal conductivity.

This paper proposes a novel three-dimensional columnar structure EBPVD coating model with contact thermal resistance between adjacent columns, in which the predicted effective thermal conductivity is in good agreement with the experimental thermal conductivity. A mathematic model of each microstructure parameter was established to reveal the relationship between parameters and the effective thermal conductivity. The heat conduction characteristics of the columnar structure coating was analyzed and the dependence of the effective thermal conductivity on microstructural parameters of the coating was studied by a finite element model as well as a mathematic model. The parameters of microstructure included the coarse column diameter, the thickness of the coating, the ratio γ of the height of fine column to coarse column and the column inclination angle. In addition, the dependence of the mechanical stress near the root of coating on microstructural parameters was investigated by a mathematical formulation, and the optimization of inclination angle of columns was proposed considering the thermal insulation and mechanical stress of the coating.

## 2. Experiments and Modeling

### 2.1. Specimen Preparation and Micrography Characterization

The columnar structure coating sample was prepared by the equipment EB-PVD 100D which was independently developed by Beihang University, and equipped with four electron guns, each with a power of 40 kw. The equipment works by focusing the high-energy electron beam on the surface of target material to make it melt quickly, evaporate and deposit on the substrate to form a coating, which has a good bond with the substrate.

The substrate of the sample was a 20 mm × 10 mm × 5 mm cuboid sample with a composition of K3 nickel-based superalloy. The composition of alloy is shown in Table 1. In order to alleviate the thermal expansion mismatch between the substrate and the ceramic coating, a bonding layer was adopted. The material of bonding layer was NiCoCrAlY, and the composition is listed in Table 2. The ceramic layer material was 8YSZ.

The preparation process was as follows: firstly, the polished substrate sample was soaked in acetone for ultrasonic cleaning. Then, a bonding layer was prepared by EB-PVD on the surface of the substrate. After bringing down to room temperature, the sample was placed in a vacuum heat treatment furnace to be heat treated at 1050 °C for 4 h. After cooling to room temperature, the surface was lightly shot blasted to make the surface of the bonding layer have a certain roughness to increase the bonding strength when the ceramic layer was deposited. Finally, the EB-PVD equipment was utilized to prepare the 8YSZ columnar structure ceramic layer coating, therein the sample rotation speed was 15 mm/min, and the substrate temperature was 950 °C.

**Figure 1:**
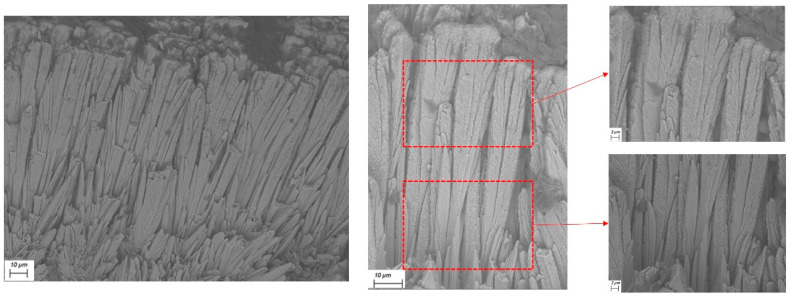
Scanning electron microscopy (SEM) micrograph of the electron beam physical vapor deposition (EB-PVD) coatings.

The cross-sectional micrography of the coating specimen was analyzed by scanning electron microscope (SEM). The classic microphotograph of the EB-PVD coating is shown in Figure 1. The coating was composed of a combination of columns, some of which touch each other, and some of which had obvious gaps among each other. Considering that the columns grew independently during the deposition process, the EB-PVD coating had obvious discontinuous characteristics in the horizontal direction.

### 2.2. Modeling and Verification

**Figure 2:**
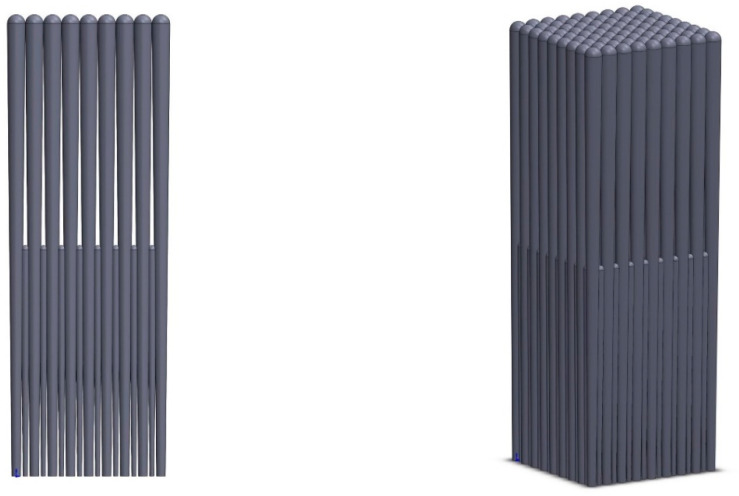
The three-dimensional columnar structure EB-PVD coating model.

According to the microstructure graph of the EB-PVD coating, the coating was composed of columns relying on each other, and there were obvious linear gaps between the columns. The shadowing effect during the vapor deposition process resulted in the typical columnar structural characteristics of the EB-PVD coating [34]. In the outer coating zone, there were columns with coarse grained structure whose head diameter were 8–12 μm and obvious gaps between the columns. In the inner coating zone, there were a number of fine-grained structure columns filling the space as much as possible, which resulted in lots of contact interface in the inner zone. A novel three-dimensional model of EB-PVD coatings was developed to describe this typical structural feature, as shown in Figure 2, with the aim to investigate the heat conduction characteristics of the columnar structure coating by utilizing commercial finite element software ANSYS.

**Figure 3:**
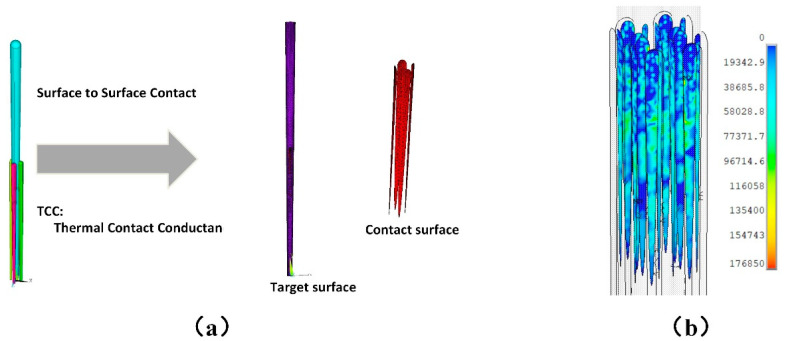
(**a**) The thermal contacting pair in simulation, and (**b**) heat flux field of the thermal contact pairs.

In this model, the columnar structure coating consisted of coarse columns and fine columns. In outer coating zone, there were obvious gaps between the coarse-grained structure columns, while the fine grained structure columns filled the space and formed many contact interfaces in the inner coating zone. The heat transfer at the contact interface between the fine columns was characterized by the thermal contact resistance, which is the additional transfer resistance when two solid surfaces in nominal contact with each other, with only contact on some discrete area elements. The heat conduction of thermal contact resistance could be expressed by the specific thermal contact pair in the finite element simulation software, which is shown in Figure 3a,b. The calculation formula of the heat flux between the contact surfaces is as follows:

(1) $$\begin{array}{c} q=\mathrm{TCC}\times \left(T_{t}-T_{c}\right) \end{array}$$

and TCC is the heat transfer coefficient of contact pair, which could be estimated by the reverse error propagation method. By adjusting the heat transfer coefficient, the error between the calculated effective thermal conductivity of the model and the experimental measured value was controlled within 5% at each level of temperature. Thereby heat transfer coefficient of contact pair was calibrated, which is 31,000 J/m^2^K in this case.

**Figure 4:**
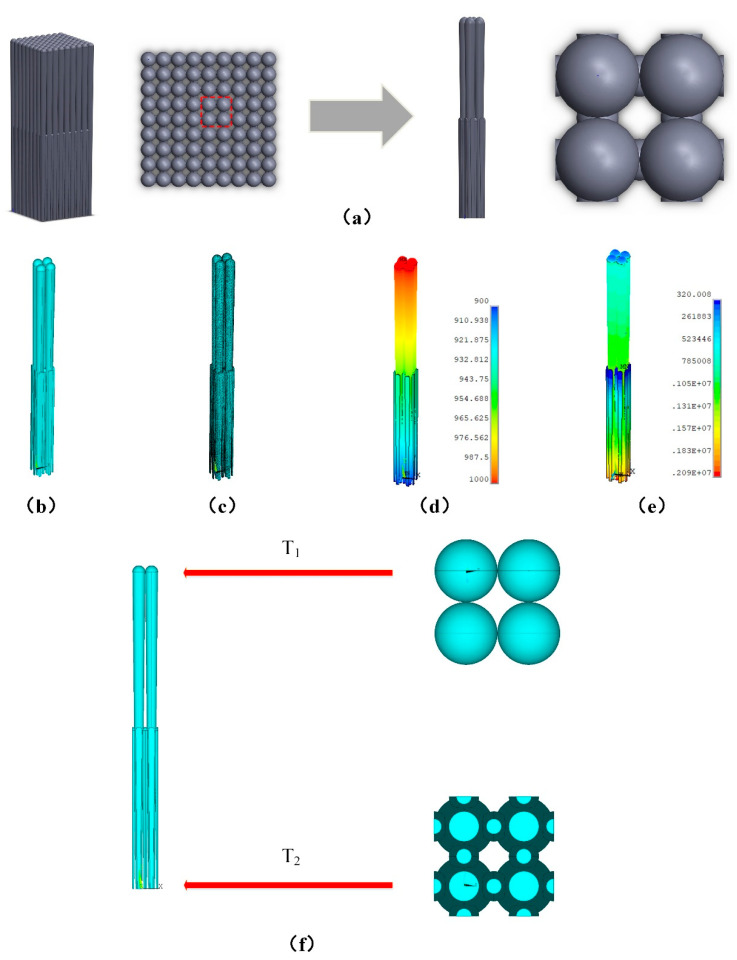
(**a**) Cell model, (**b**) geometric model, (**c**) meshed finite element model, (**d**) temperature contours of the cell model, (**e**) heat flux contours of the cell model and (**f**) boundary conditions of the cell model.

Because the model had obvious periodicity in geometry, a 2 × 2 cell model could be extracted as a cell to represent the whole model, to ensure that the geometric periodicity of the cells and the mutual energy transfer between cells were zero, as shown in Figure 4a. The cell model’s geometric model, the finite element model, the temperature contours of the model, and heat flux contours of the model are shown in Figure 4b–e, respectively. In the coating model, the height of the coarse column was 110 μm and the head diameter of coarse column was 8 μm. The finite element model had 92,782 nodes and 443,014 elements. The boundary conditions were applied as follows: a temperature load with a value of T_1_ was applied to the upper surface, a temperature load with a value of T_2_ was applied to each surface of the bottom, and the remaining surfaces were heat insulated. The details are shown in Figure 4f.

In this model, the effective thermal conductivity were computed based on Fourier conduction and heat conduction partial differential equations, as follows:

(2) $$\begin{array}{c} q=-k\nabla T \end{array}$$

(3) $$\begin{array}{c} \rho C_{p}\frac{\partial T}{\partial t}=k\nabla^{2}T \end{array}$$

Here, q is the heat flux, k is the thermal conductivity, ∇T is the temperature difference per unit thickness, ρ is the density of the coating material, $C_{p}$ is the specific heat capacity of the coating material, and $\nabla^{2}$ is the Laplace operator.

The calculation of effective thermal conductivity is based on the steady-state thermal analysis, so $\frac{\partial T}{\partial t}=0$ in the Equation (3). The temperature boundaries were given at the top and bottom surfaces, while both sides were set as insulated conditions, which established a certain temperature between the top and bottom of model. Therefore, the effective thermal conductivity can be computed with the Fourier formulation, in which the total heat flow ($Q_{s}$) between the top and bottom surfaces of the model can be obtained by the finite element numerical calculation.

(4) $$\begin{array}{c} k_{\mathrm{eff}}=\frac{Q_{s}\times h}{A_{s}\times ∆T} \end{array}$$

where $Q_{s}$ is the total heat flow which is going through the top to the bottom of the model, $A_{s}$ is the area of the top surface of the model, h is the coating thickness and ∆T is the temperature difference.

**Figure 5:**
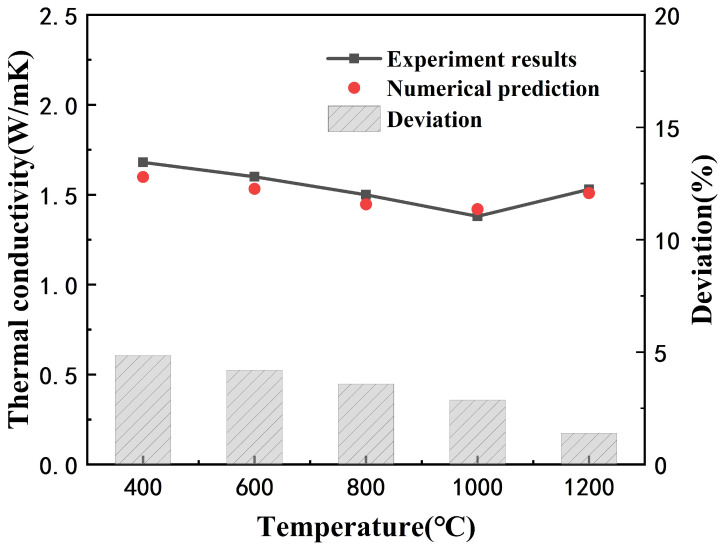
Comparison of numerical results and experimental results.

The comparative results between the predicted value of the effective thermal conductivity and the experimental measured value of the coating [35] are shown in Figure 5, which show a good agreement.

## 3. Results and Discussion

### 3.1. The Effect of Coarse Column Diameter on Effective Thermal Conductivity

**Figure 6:**
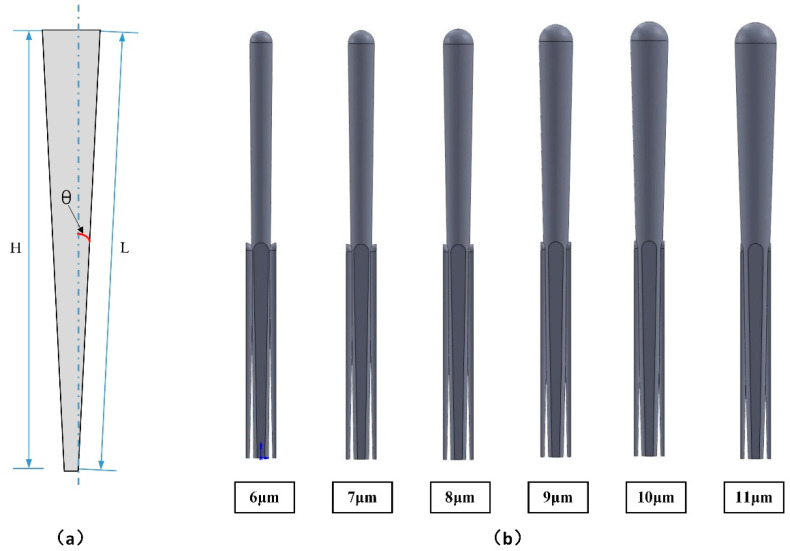
(**a**) The sectional view of coarse column and (**b**) coating models with different coarse column diameter.

Firstly, the analytical model of the coarse column diameter was set up to reveal the relationship between coarse column diameter and thermal conductivity. The taper θ was defined as the ratio of the radius to the height of the coarse column in Equation (5). The dimensionless quantity S was defined to measure the longest heat transfer path per unit thickness of the coating model, and the function relationship between the taper θ and the heat transfer path per unit thickness is established in Equation (6). These variables were described in the sectional view of the coarse column in Figure 6a.

(5) $$\begin{array}{c} \theta =\tan^{-1}\frac{d_{b}}{2H} \end{array}$$

(6) $$\begin{array}{c} S=\frac{L}{H}=\frac{1}{\cos\theta }=\sec\theta \end{array}$$

**Figure 7:**
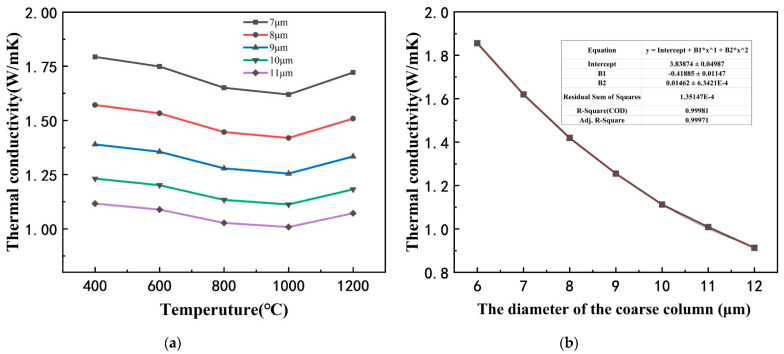
Effective thermal conductivity of models with different coarse column diameter of the coating: (**a**) at different levels of temperature, (**b**) at 1000 °C.

Then, the effect of coarse column diameter on effective thermal conductivity was studied by the finite element model. Keep other variables constant, change the diameter of the coarse column, and the models with different coarse column diameter are shown in Figure 6b. The calculation results of the effective thermal conductivity are presented in Figure 7, in which there was a decline of the effective thermal conductivity as the diameter of the coarse column increased. Performing polynomial fitting on the curve in Figure 7b, the function of effective thermal conductivity and column head diameter could be obtained, as shown in the following Formula (7). The specific fitting parameters are shown in Figure 7b.

(7) $$\begin{array}{c} k_{\mathrm{eff}}=3.8387-0.4189d+0.0146d^{2} \end{array}$$

According to the Equations (5) and (6), the taper θ increases with increasing the diameter of the coarse column head, resulting in a longer heat transfer path per unit thickness of the coating, which makes the effective thermal conductivity decrease.

### 3.2. The Effect of the Thickness of Coatings on Effective Thermal Conductivity

**Figure 8:**
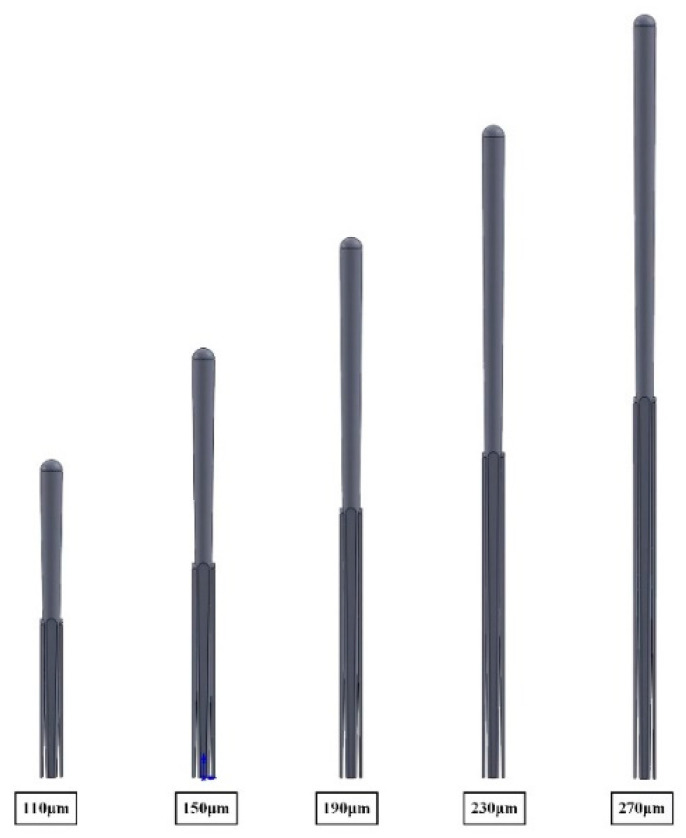
Coating models with different thickness of coatings.

**Figure 9:**
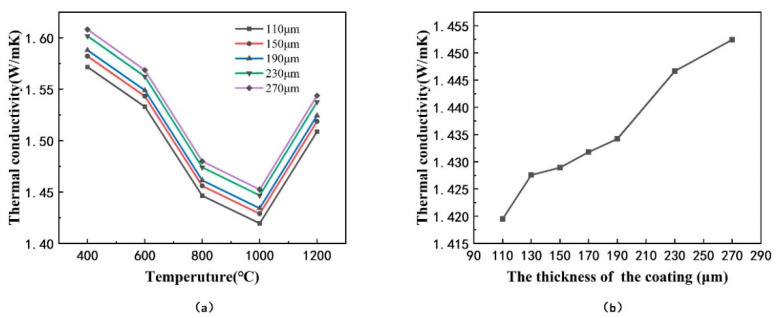
Effective thermal conductivity of models with different thickness of coatings: (**a**) at different levels of temperature, (**b**) at 1000 °C.

Based on the typical finite element model of the columnar structure coating, the effect of the thickness of coating on effective thermal conductivity was investigated. Keep the other variables fixed, change the height of the coarse columns, and the models with different coating thickness are presented in Figure 8. The calculation results of effective thermal conductivity are provided in Figure 9. The results showed that the effective thermal conductivity increased as the coating thickness increased, which is consistent with the experimental results in literature [24]. According to the Equations (5) and (6), the taper θ of the column decreased as the thickness of the coating increased, resulting in a shorter heat transfer path per unit thickness of the coating, which made the effective thermal conductivity increase.

### 3.3. The Effect of the Ratio γ of the Height of Fine Column to Coarse Column on Effective Thermal Conductivity

**Figure 10:**
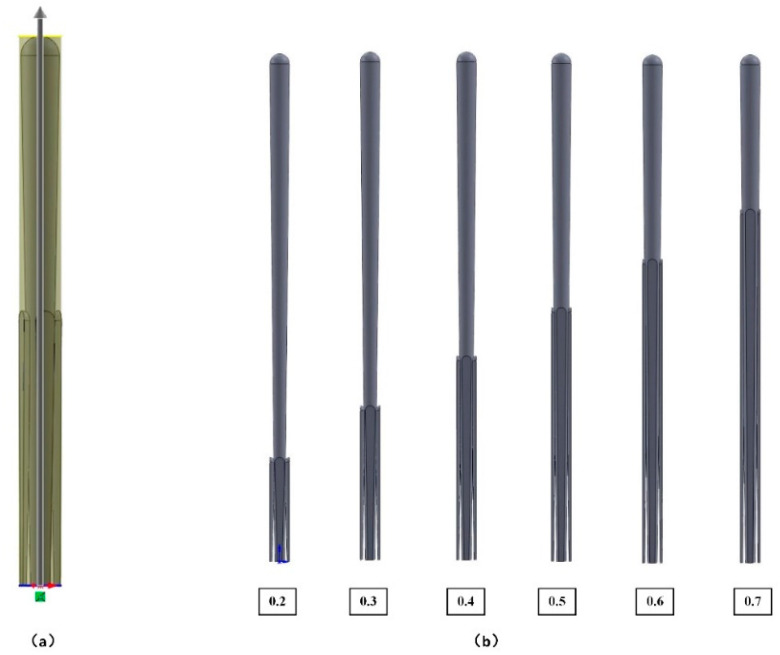
(**a**) Calculation diagram of porosity *p* and (**b**) coating models with different ratio γ.

First, a mathematical model of the porosity of coating and microstructure parameters ($h_{c}$, $d_{c}$, $h_{f}$, $d_{f})$ was established which could predict thermal conductivity. The porosity *p* was calculated by the ratio of the volume of pores to the whole volume of model, which is shown in Figure 10a and following equations.

(8) $$p=1-\frac{V_{c}+V_{f}}{V_{w}}$$

(9) $$V_{f}=2\left[\frac{1}{3}{\pi h}_{f}{\left(\frac{d_{f}}{2}\right)}^{2}+\frac{2}{3}\pi {\left(\frac{d_{f}}{2}\right)}^{3}-\frac{1}{3}{\pi h}_{f0}{\left(\frac{d_{f0}}{2}\right)}^{2}\right]$$

(10) $$\begin{array}{c} V_{w}=A_{w}h_{w}=\pi {\left(\frac{d_{c}}{2}\right)}^{2}\left(h_{c}+\frac{d_{c}}{2}\right) \end{array}$$

(11) $$\begin{array}{c} p\sim f\left(h_{c}、d_{c}、h_{f}、d_{f}\right) \end{array}$$

where $V_{c}$ is the volume of the coarse column, $V_{f}$ is the volume of the fine column, $h_{c}$ is the height of the coarse column,${\;d}_{c}$ is the diameter of the coarse column, $h_{f}$ is the height of the fine column, $d_{f}$ is the diameter of the fine column, $h_{c0}$, $h_{f0}$ are the height of the taper tip assumed in the cone volume calculation process. Because of $h_{c0}$<<$h_{c},h_{f0}$<<$h_{f}$, the formulas can be simplified as follows.

(12) $$V_{c}=\frac{\pi }{12}\left(h_{c}{d_{c}}^{2}+{d_{c}}^{3}\right)$$

(13) $$V_{f}=\frac{\pi }{6}\left(h_{f}{d_{f}}^{2}+{d_{f}}^{3}\right)$$

(14) $$\begin{array}{c} p=1-\frac{h_{c}{d_{c}}^{2}+{d_{c}}^{3}+2h_{f}{d_{f}}^{2}+2{d_{f}}^{3}}{3{d_{c}}^{2}\left(h_{c}+\frac{d_{c}}{2}\right)}\sim f\left(d_{c},h_{c},d_{f},h_{f}\right) \end{array}$$

**Figure 11:**
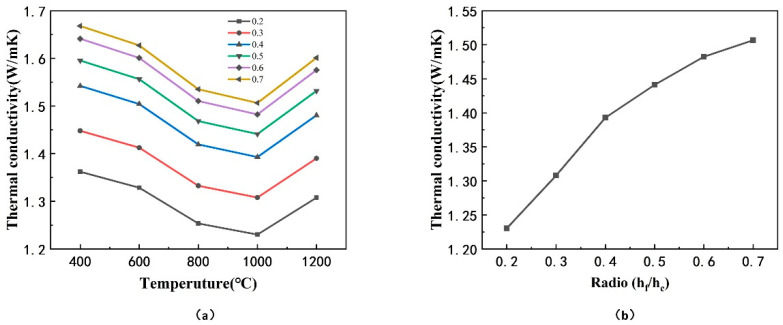
Effective thermal conductivity of models with different ratio γ of the coating: (**a**) at different levels of temperature, (**b**) at 1000 °C.

Then, the dependence of the effective thermal conductivity on the ratio γ of the height of fine column to coarse column was studied based on the finite element model of coating. Keep the thickness of the coating and the diameter of the coarse column constant, change the ratio γ, and the models with different ratio γ are presented in Figure 10b. The calculated results of the effective thermal conductivity are shown in Figure 11, in which there is an increase of the effective thermal conductivity as the ratio γ of coating increases.

According to the analysis of mathematical model Equation (14) of the porosity of the coating, the porosity decreased linearly as the height of the fine column $\;h_{f}$ increased, which caused the effective thermal conductivity to increase. As the ratio of the height of fine column to coarse column increased, a greater volume of fine columns filled in the gap between the coarse columns and the discontinuity in the horizontal direction of the coating decreased, thereby increasing the effective thermal conductivity [31].

### 3.4. The Effect of the Inclination Angle α on Effective Thermal Conductivity

The inclination angle of the entire columns of the coating could be controlled by adjusting the process parameters [36]. However, the influence of the inclination angle on the thermal insulation performance of the coating was still unclear, which will be revealed in this part.

**Figure 12:**
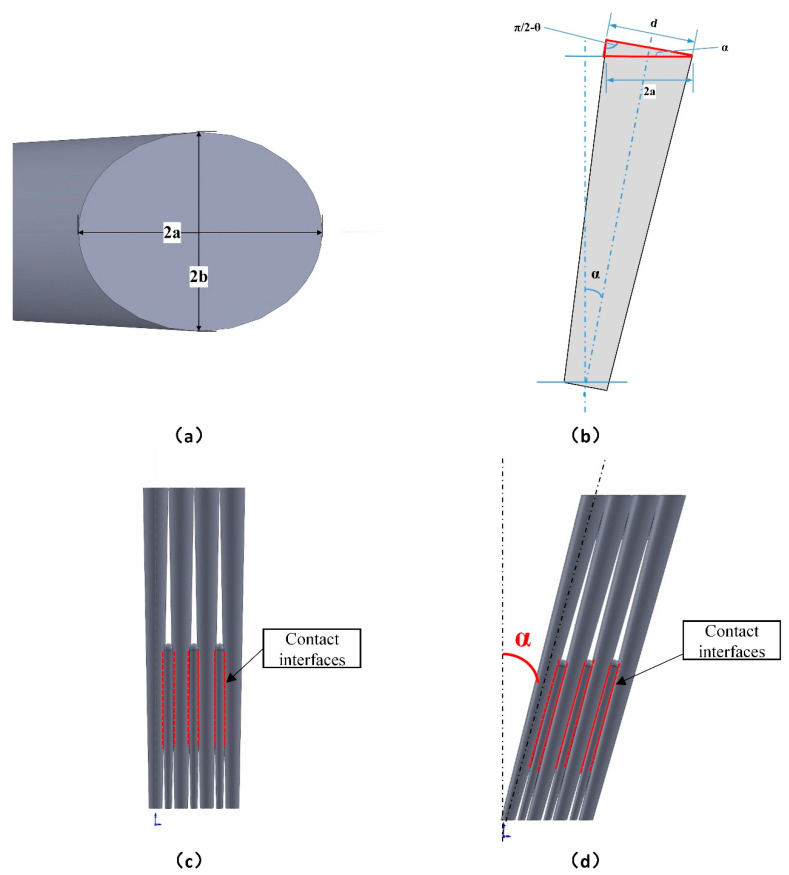
(**a**) Top view of the coating with inclined column, (**b**) the cross-sectional view of the inclined coating, (**c**) coating model with inclination angle 0°, and (**d**) coating model with inclination angle α.

First, a mathematical model of the inclination angle α was established to predict the effective thermal conductivity. As mentioned above, the thermal conductivity is calculated as Formula (15), where $A_{S}$ is the area of the coating’s top surface which is elliptical as described in Figure 12a.

(15) $$k_{\mathrm{eff}}=\frac{Q_{s}\times h}{A_{s}\times ∆T}$$

(16) $$\begin{array}{c} A_{S}=\pi \times a\times b \end{array}$$

Herein b is the length of the semiminor axis of the ellipse, and a is the length of the semimajor axis which is obtained by analyzing the cross-sectional view of the coating, as shown in Figure 12b.

(17) $$\begin{array}{c} 2a=d\cos\alpha +d\sin\alpha \tan\left[\left(\frac{\pi }{2}-\theta \right)-\left(\frac{\pi }{2}-\alpha \right)\right]=d\cos\alpha +d\sin\alpha \tan\left(\alpha -\theta \right) \end{array}$$

(18) $$\begin{array}{c} \theta =\tan^{-1}\frac{d}{2h} \end{array}$$

The taper θ << α, so tan (α-θ) is approximately equal to tanα, and Equation (17) can be simplified as Equation (19). The mathematic model of the inclination angle α and effective thermal conductivity $k_{\mathrm{eff}}$ was set up as Formula (20).

(19) $$\begin{array}{c} 2a=\;d\cos\alpha +d\sin\alpha \tan\alpha =\frac{d}{\cos\alpha } \end{array}$$

(20) $$\begin{array}{c} k_{\mathrm{eff}}=\frac{Q_{s}\times h}{\frac{{\pi d}^{2}}{4\cos\alpha }\times ∆T}=\frac{4Q_{s}h\cos\alpha }{{\pi d}^{2}∆T}\sim f\left(\alpha \right) \end{array}$$

**Figure 13:**
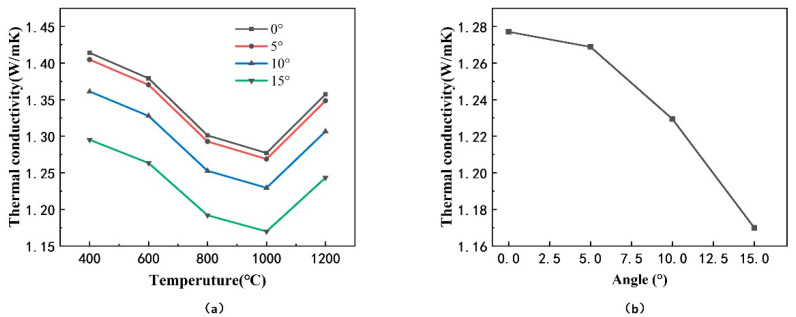
Effective thermal conductivity of models with different inclination angle α of the coating: (**a**) at different levels of temperature, (**b**) at 1000 °C.

Then, based on the finite element model of coating, the dependence of the effective thermal conductivity of TBCs on the inclination angle α was studied. Keep other parameters fixed, change the inclination angle α of the overall column, and the models with different inclination angle α are presented in Figure 12d. The calculation results of the effective thermal conductivity are provided in Figure 13, in which there is a decline of the effective thermal conductivity as the inclination angle of the column increases.

It can be deduced from the mathematic model of the inclination angle in Formula (20) that the effective thermal conductivity was a function of cosα, which decreased with increasing of angle α, when α was in the range from 0 to π/2. It could be explained from the analyzed model of the heat transfer path in Formula (6) that the heat transfer path per unit thickness increased with increase of the inclination angle, which led to a decrease of effective thermal conductivity. In addition, the angle between the contact interfaces of inter-columns and the longitudinal heat flow increased with the increasing inclination angle of the columns, resulting in a longer heat transfer path when the longitudinal heat flow passed the interfaces.

### 3.5. The Effect of Microstructural Parameters on Mechanical Stress

The effective thermal conductivity decreased with increasing inclination angle of the columns of the coating, which might influence the interfacial stress between the coating and the substrate. The pressure on top of the column would make the column bear an additional bending moment to make the root of column bear tensile stress. So, a mathematic model of the impact of microstructural parameters on the mechanical stress of the column was built to optimize the microstructure of the coating with the constraints by mechanical stress.

**Figure 14:**
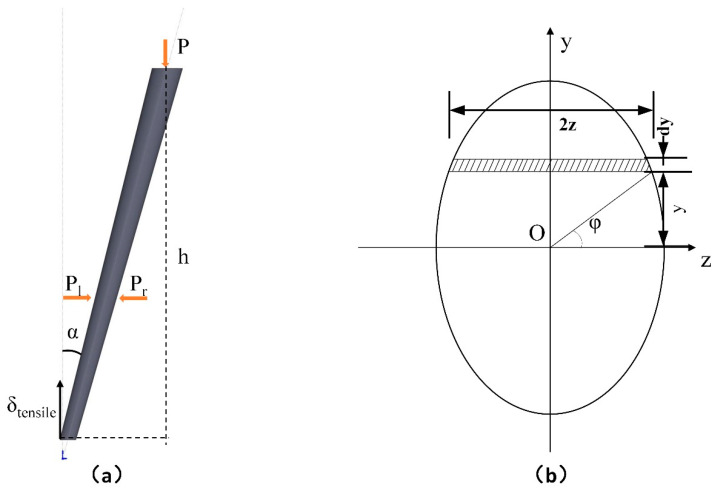
(**a**) The pressure load of the column, and (**b**) the calculation of $I_{z}$ of the elliptical shape of the bottom section.

As shown in Figure 14a, the top of the column bore an external pressure, the left bore the pressure from the left column, and the right bore the pressure from the right column. The left and right forces could be considered to cancel each other out. Therefore, the column would only bear a pressure at the top, which makes the root of the column bear a bending moment when the inclination angle of the column is α.

The computation of the normal stress at any point on the cross-sectional area of the root of column is shown in Equation (21), when the column is subjected to a bending moment.

(21) $$\begin{array}{c} \sigma =\frac{M\times y}{I_{z}} \end{array}$$

(22) $$\begin{array}{c} M=F\times l_{m} \end{array}$$

(23) $$\begin{array}{c} F=PA_{s} \end{array}$$

where σ is the stress, M is the bending moment, $I_{z}$ is the moment of inertia of section to neutral axis z, p is the external pressure on the top of the coating and $A_{s}$ is the area of the top surface of the column, which is computed by Equation (16).

The formula for calculating $I_{z}$ of the elliptical shape of the bottom section can be described in Figure 14b and Equation (24):

(24) $$\begin{array}{c} I_{z}=\overset{\;}{\underset{A}{\int }}y^{2}\mathrm{dA}=\frac{{\pi b}_{1}{a_{1}}^{3}}{4} \end{array}$$

(25) $$\begin{array}{c} a_{1}=\frac{d_{b}}{2\cos\alpha } \end{array}$$

(26) $$\begin{array}{c} b_{1}=\frac{d_{b}}{2} \end{array}$$

Here, $a_{1}$ is the length of semimajor axis of the elliptical section in bottom of the column, $b_{1}$ is the length of semiminor axis of the elliptical section in bottom of the column, and $d_{b}$ is the diameter of the root of the column when it is vertical.

The place where the stress is maximum in the section is at y_max_ = a_1_, so;

(27) $$\begin{array}{c} \sigma_{\max}=\frac{M\times y_{\max}}{I_{z}}=\frac{M\times a_{1}}{I_{z}}=\frac{8{{\mathrm{hd}}_{c}}^{2}P\sin\alpha }{{d_{b}}^{3}} \end{array}$$

(28) $$\begin{array}{c} \xi =\frac{8{{\mathrm{hd}}_{c}}^{2}}{{d_{b}}^{3}} \end{array}$$

(29) $$\begin{array}{c} \sigma_{\max}=\xi P\sin\alpha \end{array}$$

where ξ is defined as rectangle factor of the column and ${\;d}_{c}$ is the diameter of the head of the column when it is vertical.

The mathematic model was established to analyze the influence of microstructural parameters on the mechanical stress of the column, as shown in Formula (27), in which $d_{b}$ is the diameter of the root of the column and could be regarded as constant, since it is difficult to adjust during the coating deposition process. The effect of the coarse column diameter, the coating thickness and inclination angle on the mechanical stress of the column was analyzed by the mathematical model, respectively.

The partial differential equation of $f\left(d_{c},h,\alpha ,P\right)$ to the coarse column diameter d_c_ is as follows:

(30) $$\begin{array}{c} f_{d_{c}}^{\prime }=\frac{\partial \sigma_{\max}}{\partial d_{c}}=\frac{16\mathrm{hP}\sin\alpha }{{d_{b}}^{3}}d_{c} \end{array}$$

The mechanical stress increases with the coarse column diameter increasing, as $f_{d_{c}}^{\prime }$ is positive when α is in the range from 0 to π/2.

The partial differential equation of $f\left(d_{c},h,\alpha ,P\right)$ to the thickness of the coating h is as follows:

(31) $$\begin{array}{c} f_{h}^{\prime }=\frac{\partial \sigma_{\max}}{\partial h}=\frac{8{{\mathrm{Pd}}_{c}}^{2}\sin\alpha }{{d_{b}}^{3}} \end{array}$$

The mechanical stress increases with the thickness of coating increasing, as $f_{h}^{\prime }$ is positive when α is in the range from 0 to π/2.

The partial differential equation of $f\left(d_{c},h,\alpha ,P\right)$ to the inclination angle α is as follows:

(32) $$\begin{array}{c} f_{\alpha }^{\prime }=\frac{\partial \sigma_{\max}}{\partial \alpha }=\frac{8{{\mathrm{Phd}}_{c}}^{2}}{{d_{b}}^{3}}\cos\alpha \end{array}$$

The mechanical stress increases with the inclination angle of the column increasing, as $f_{\alpha }^{\prime }$ is positive when α is in the range from 0 to π/2. Previous studies have shown that the effective thermal conductivity decreases as the inclination angle α increases. Accordingly, in the optimization design of the inclination angle of the coating structure, in order to obtain the lowest effective thermal conductivity and ensure that the coating is not damaged, $\sigma_{\max}$ should be equal to the maximum load [$\sigma_{m}$] that the material can withstand. Ultimately, the optimal critical value of the inclination angle α is as follows.

(33) $$\begin{array}{c} \alpha_{\max}=\sin^{-1}\frac{\left(\sigma_{m}\right)}{\xi P} \end{array}$$

## 4. Conclusions

The effect of microstructural parameters on the effective thermal conductivity of the EB-PVD thermal barrier coating was investigated considering the constraint by mechanical stress. The conclusions could be drawn as follows:

(1) Increasing the inclination angle α (from 0° to 15°) of the columns could result in a decline of effective thermal conductivity (from 1.277 W/mK to 1.169 W/mK, at 1000 °C) and an increase of the mechanical stress at the root of the coating. The optimization of the inclination angle of the coating is proposed as:
  $$\alpha_{\max}=\sin^{-1}\frac{\left(\sigma_{m}\right)}{\xi P}$$
(2) Increasing the diameter of the coarse column could result in a decline of effective thermal conductivity, and an increase of the mechanical stress at the root of the coating when the columns of the coating is inclined. The function of effective thermal conductivity and column head diameter are shown in the following formula:
  $$k_{\mathrm{eff}}=3.8387-0.4189d+0.0146d^{2}$$
(3) Increasing the thickness of the coating would lead to an increase of the effective thermal conductivity, and an increase of the mechanical stress at the root of the coating when the columns of the coating are inclined.
(4) Increasing the ratio γ (from 0.2 to 0.7) of the height of fine column to coarse column in the coating would make the effective thermal conductivity (from 1.231 W/mK to 1.507 W/mK, at 1000 °C) increase, since the porosity decreases and the discontinuity in the horizontal direction of the coating decreases.

## Figures and Tables

**Table 1:** The alloy composition of K3 nickel-based superalloy.

Element	Ni	Cr	Co	Al	W	Mo	Ti	Fe
wt. %	Bal.	10.0–12.0	4.5–6.0	5.3–5.9	4.8–5.5	3.8–4.5	2.3–2.9	≤2.0

**Table 2:** The composition of NiCoCrAlY.

Element	Ni	Co	Cr	Al	Y
wt. %	49	20	22	8	1

## Data Availability

Data sharing is not applicable to this article.

## Nomenclature

k	thermal conductivity
$k_{\mathrm{eff}}$	effective thermal conductivity
∇T	temperature difference per unit thickness
ρ	density of the coating material
$C_{p}$	specific heat capacity of the coating material
$Q_{s}$	total heat flow
$A_{s}$	area of the top surface of the model
∆T	temperature difference
θ	taper of the coarse column
S	the longest heat transfer path per unit thickness
γ	ratio of the height of fine column to coarse column
p	porosity of the coating
$h_{c}$	height of the coarse column
$d_{c}$	diameter of the coarse column
$h_{f}$	height of the fine column
$d_{f}$	diameter of the fine column
α	inclination angle of the entire columns
σ	stress of the root of column
M	bending moment
$I_{z}$	moment of inertia of section to neutral axis z
p	external pressure on the top of the coating
$d_{b}$	diameter of the root of the column
ξ	rectangle factor of the column
